# Efficacy of antiseptics in a novel 3-dimensional human plasma biofilm model (hpBIOM)

**DOI:** 10.1038/s41598-020-61728-2

**Published:** 2020-03-16

**Authors:** M. Besser, M. Dietrich, L. Weber, J. D. Rembe, E. K. Stuermer

**Affiliations:** 1 Institute for Translational Wound Research, Centre for Biomedical Education and Research (ZBAF), Witten/Herdecke University, Witten, Germany; 2 Department of Vascular Medicine, University Heart Center, Translational Wound Research, University Medical Center Hamburg-Eppendorf, Hamburg, Germany

**Keywords:** Antimicrobial resistance, Translational research

## Abstract

The increasing incidence of non-healing wounds constitutes a pivotal socio-economic burden. 60–80% of chronic wounds are colonized by pathogenic microorganisms within a protective extracellular polymeric substance, bearing a great challenge in wound management. Human plasma was used to prepare the biofilm model (hpBIOM), adding pathogens to the plasma and forming Coagula-like discs with integrated pathogens were produced. The antiseptics Octenisept and Lavasorb were tested regarding their antibacterial properties on clinically relevant biofilm-growing bacteria (MRSA, *P. aeruginosa*) in the hpBIOM. Biofilm-typical glycocalyx-formation was confirmed using immunohistochemical staining. Treatment of a 12 h-maturated biofilm with Octenisept resulted in complete eradication of *P. aeruginosa* and MRSA after 48 h. Lavasorb proved less effective than Octenisept in this setting. In more mature biofilms (24 h), both antiseptics showed a delayed, partially decreased efficacy. Summarized, the hpBIOM provides essential factors for a translational research approach to be used for detailed human biofilm analyses and evaluation of antimicrobial/-biofilm properties of established and novel therapeutic strategies and products. Octenisept and Lavasorb showed an attenuated efficacy in the hpBIOM compared to planktonic conditions and previously published biofilm-studies, prompting the question for the necessity of introducing new international standards and pre-admission requirements on a translational base.

## Introduction

Due to demographic changes, the incidence of chronic wound development in the population increases constantly. Chronic wounds are mostly of multi-factorial origin deriving from underlying diseases such as disturbed blood perfusion due to arterial or venous insufficiencies or in the context of diabetes^1^. Disturbance of regular blood circulation and concomitant tissue necrosis pave the way for bacterial attachment, growth, colonisation, and infection^2,3^. Indeed, microscopic analyses revealed, that 60–80% of chronic wounds are colonized by densely arranged mono- and polymicrobial communities, comprised of bacterial and/or fungal microorganisms. These microorganisms produce and reside within an extracellular polymeric substance (EPS)^4–6^. Various studies postulate, that persisting biofilms on chronic wounds negatively affect cellular behaviour in tissue repair processes, inflammatory cellular response and the innate immune system^7,8^. Biofilm-growing microorganisms have developed a great variance of survival strategies, e.g. a slower growth rate, adapted stress response, increased transfer of antimicrobial resistance genes and due to the EPS, a limited penetration of antibiotics and other biocides^9–14^. Because of the increasing antimicrobial resistance, extensive surgical tissue debridement is necessary, for mechanical biofilm disruption and wound cleansing. Besides the distress and pain potentially caused to the patient, an increase in wound size and depth has to be accepted initially, while the wound may still fail to progress to healing^15^.

Based on the importance of controlling biofilms in the management of chronic wounds and the threat posed to unimpaired wound healing, basic research to understand human wound-biofilm formation, organization and maintenance as well as the development and proper evaluation of therapeutic strategies is of most importance. To address this need, appropriate model systems for *in-vitro* analyses are mandatory. However, the general term “biofilm model” is not yet consistently defined. Solutions consisting of planktonic bacteria in growth-promoting artificial medium are used as *in-vitro* ‘biofilm models’^16–20^. Further systems were developed and applied as *in-vitro* biofilm models, usually based on the attachment and growth of bacteria on adhesive materials, e.g. plastic wells, glass slides or different medical devices^21,22^. Some authors described skin explants (abdominoplastic or animal skin) or multi-layered cultivated keratinocytes colonised with bacteria or bacteria-conditioned medium, as biofilm model^21,23–26^. Two 3-dimensional *in-vitro* models have been previously described: One based on a liquid-filled chamber^22,27,28^, the other comprised of a collagen matrix with serum-proteins^29^. Unfortunately, all these models show important limitations for a translation of results into clinical wound care routine. These factors include bacteria being cultivated in specifically growth-promoting nutrient media, with the lack of a human physiological wound-microenvironment or the representation of the human immune competency.

For this study we used a newly described human plasma biofilm model (hpBIOM), which was recently developed and published by our working group to specifically mimic a human, biofilm-challenged wound environment avoiding the deficiencies described above^30^. The aim was to primarily observe two established antiseptics in the newly developed challenging human biofilm environment with regard to their antiseptic performance against two commonly encountered biofilm-forming pathogens (MRSA, *P. aeruginosa*) after 12 and 24 hours of biofilm maturation in the hpBIOM.

## Methods

### Bacterial strains

*Pseudomonas aeruginosa* (DSM no. 939) was obtained from the Leibniz-Institute DSMZ- German Collection of Microorganisms and Cell Cultures. Methicillin-resistance *Staphylococcus aureus* (MRSA) was a clinical isolate, kindly provided by B. Ghebremedhin (Helios university medical centre, Wuppertal, Germany). Both strains were cultured on casein/soy peptone agar plates (CSA; pH 7.2) containing 15 g/l casein peptone, 5 g/l soy peptone, 5 g/l sodium chloride and 15 g/l agar (AppliChem, Darmstadt, Germany) under aerobic conditions at 37 °C.

### Human plasma biofilm model (hpBIOM) preparation

Plasma preserves and buffy coats from anonymous donors were obtained from the DRK-Blutspendedienst West (Hagen, Germany). First, the buffy coat was transferred to a 50 ml reaction tube (Sarstedt AG & Co., Nürnbrecht, Germany) and centrifuged at 3000 rpm for 30 min at room temperature (RT) to separate remaining erythrocytes. The resulting supernatant of blood plasma and layer of immunocompetent cells was collected and added to the plasma preserve of the same donor in a sterile glass bottle. The resulting mixture of plasma and buffy coat was gently mixed and continuously shaken at 100 rpm at 22 °C. Subsequently, a bacterial solution of the individual test pathogen was prepared by colony-picking and the solution adjusted to a 0.1 McFarland standard. For final preparation a corresponding amount of prepared bacterial solution was added to the plasma/buffy coat mixture to result in initial pathogen concentration of 2*10^6^ cfu per 3 ml plasma solution, creating a master mixture of plasma, buffy coat and pathogen. To induce fibrin polymerization (for disc-formation), 18.3 µl CaCl_2_ (500 mM) per ml plasma was added to the master mixture. Finally, to prepare individual hpBIOMs, 3 ml master mixture per well were immediately transferred into 6-well culture plates (Sarstedt AG & Co., Nürnbrecht, Germany). The plates were incubated for at least 12 h on a rotation shaker at 50 rpm at 37 °C.

### Histomorphological investigation of the hpBIOM structure

To asses biofilm-formation, glycokalyx and EPS deposition as well as the molecular structure, several histomorphological and immunohistochemical analyses of the untreated hpBIOM were performed.

### Gram-staining of the hpBIOM

After 12 h, the biofilm model was transferred to a 15 ml reaction tube and fixed in 4% paraformaldehyde at 4 °C overnight, followed by overnight incubation in 30% (w/v) sucrose for dehydration (all AppliChem GmbH, Darmstadt, Germany). The biofilms were embedded in Tissue-Tek O.C.T Compound (Science Services, Munich, Germany) on dry ice. Cryosections were prepared with 12 *μ*m thickness (Cryostat, Leica Microsystems AG, Wetzlar, Germany) and stored at −80 °C. For Gram-staining, the sections were placed in aqua dest. and incubated with crystal violet solution for 10 sec. After brief washing with aqua dest., the slides were rinsed with Gram’s iodine solution for 10 sec., briefly washed again with aqua dest. and decolorized by acetone incubation for 2 sec. After another washing step, the sections were counterstained with safranin for 15 sec., dehydrated in absolute alcohol and mounted in Thermo Scientific Shandon Immu-Mount (Fisher Scientific, Schwerte, Germany).

### Scanning electron microscopy (SEM) of the hpBIOM

The hpBIOMs were fixed with 0.1 M cacodylate buffer (2.5% glutaraldehyde, 2% polyvinylpyrrolidone and 75 mM NaNO_2_) for 1 h at 4 °C. Samples were rinsed with 0.1 M cacodylate buffer without glutaraldehyde. Freeze fracture samples of the biofilms were prepared by freezing in liquid nitrogen and subsequent fragmentation. The glycocalyx staining was performed in a solution comprised of 2% arginine-HCl, glycine, sucrose and sodium glutamate for 18 h at RT. Afterwards, biofilm models were washed in distilled water and immersed in a mixture of 2% tannic acid and guanidine-HCl each for 5.5 h at RT, followed by another washing step with distilled water and incubation in a 1% OsO_4_ solution for 30 min at RT. After three more rinsing steps with distilled water, the specimens were dehydrated, dried in liquid CO_2_, sputtered with gold palladium and finally examined with a Zeiss Sigma SEM (Zeiss, Oberkochen, Germany) using 2 kV acceleration voltage and an in-lens detector.

### Immunohistochemical investigation of the hpBIOM

For the immunohistochemical investigation of the glycokalyx, the Concanavalin-FITC labeled antibody (Con A-FITC) (Sigma, Saint louis, Missouri, USA) was used (50 µg/ml in PBS/0.1% Triton). Nuclei were stained with the SYTO red fluorescent nucleic acid staining (Fisher Scientific, Schwerte, Germany) by adding 20 µM SYTO dye to the Con A-FITC incubation buffer. This solution was added to the slice and incubated for 30 min at RT, followed by 3 washing steps in PBS. The slides were mounted in Thermo Scientific Shandon Immu-Mount (Fisher Scientific, Schwerte, Germany).

### Histomorphological investigation of the hpBIOM: Scanning electron microscopy (SEM)

The hpBIOMs were fixed with 0.1 M cacodylate buffer (2.5% glutaraldehyde, 2% polyvinylpyrrolidone and 75 mM NaNO_2_) for 1 h at 4 °C. Samples were rinsed in 0.1 M cacodylate buffer without glutaraldehyde. The biofilm discs were divided into small pieces by freezing in liquid nitrogen. The glycokalyx staining was performed in a solution comprised of 2% arginine-HCl, glycine, sucrose and sodium glutamate for 18 h at RT. The biofilms were washed in distilled water followed by immersion in a mixture of each 2% tannic acid and guanidine-HCl for 5.5 h at RT. The samples were rinsed again in distilled water and incubated in a 1% OsO_4_ solution for 30 min at RT. After three rinsing steps with distilled water the specimens were dehydrated, dried in liquid CO_2_, sputtered with gold palladium and finally examined with a Zeiss Sigma SEM (Zeiss, Oberkochen, Germany) using 2 kV acceleration voltage and an inlens detector.

### Time-kill-assay

Two antiseptics were tested using a time-kill-assay investigating their antimicrobial efficacy on biofilm-growing bacteria: Octenisept (0.1% octenidine-dihydrochloride/2% phenoxyethanol; Schülke & Mayr GmbH, Norderstedt, Germany) and Lavasorb (0.04% polyhexanide; Fresenius Kabi Deutschland GmbH, Bad Homburg, Germany). For this analysis, hpBIOMs with MRSA or *P. aeruginosa* were prepared in 6-well culture plates (Sarstedt AG & Co., Nürnbrecht, Germany). After either 12 h or 24 h of undisturbed biofilm maturation, biofilm models were treated with 600 µl of either antiseptic solution and incubated on a rotation shaker (50 rpm) at RT. Every 24 h, the same dosage of antiseptic solution was re-administered (mimicking a daily antiseptic treatment in clinical routine). Controls were treated with PBS only. After 6, 24, 48 and 72 h of exposure, antiseptic activity was terminated using a neutralization buffer containing 3 g/l sodium thiosulfate, 30 g/l polysorbate 80 (Tween 80) and 3 g/l lecithin (all Carl Roth GmbH u. Co. KG, Karlsruhe, Germany). Previously performed validations confirmed no antimicrobial or disintegrative effect on the biofilm model as well as sufficient neutralizing capability of the neutralization buffer.

### Quantification of bacterial growth and survival

After completed antiseptic exposure, biofilm models were dissolved by adding 3 ml (1:1 v/v) of a 10% (w/v) bromelain solution (Bromelain-POS, RSAPHARM Arzneimittel GmbH, Saarbrücken, Germany) in PBS. The discs were detached from the well margins and punctured for improved permeability for enzymatic digestion. Completely dissolution was achieved within 2 h at 37 °C. For quantification of surviving bacteria serially tenfold dilutions were prepared and 100 µl aliquots plated on CSA plates and incubated at 37 °C overnight. The bacterial burden (cfu/ml) was determined using a computerized colony counter (Scan 500, Interscience, France)

### Statistical analysis

Due to the donor-specific plasma composition, results were analyzed separately. Experiments were performed in triplicates per donor for each pathogen and data analyzed using GraphPad Prism 6 (GraphPad Software, Inc., La Jolla, USA). Two-way ANOVA followed by Tukey´s HSD test as post-hoc evaluation for multiple comparisons was applied as statistical analysis. A p-value of *p* ≤ 0.05 was considered statistically significant. (*p ≤ 0.05; **p ≤ 0.01; ***p ≤ 0.001). (Supplementary Tables).

### Consent for publication

All authors give consent for publication

## Results and Discussion

**Figure 1:**
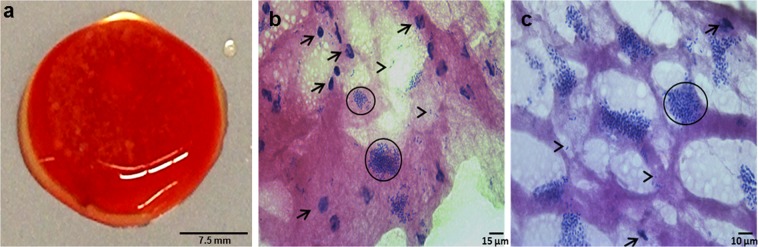
Morphology of the hpBIOM. (**a**) Photomicrograph of a 12 h-maturated biofilm disc in surface-view. **(b**,**c)** Gram-staining of a MRSA hpBIOM. Arrows indicate human cells, arrow heads depict single bacteria or small colonies. Large colonies are circled.

**Figure 2:**
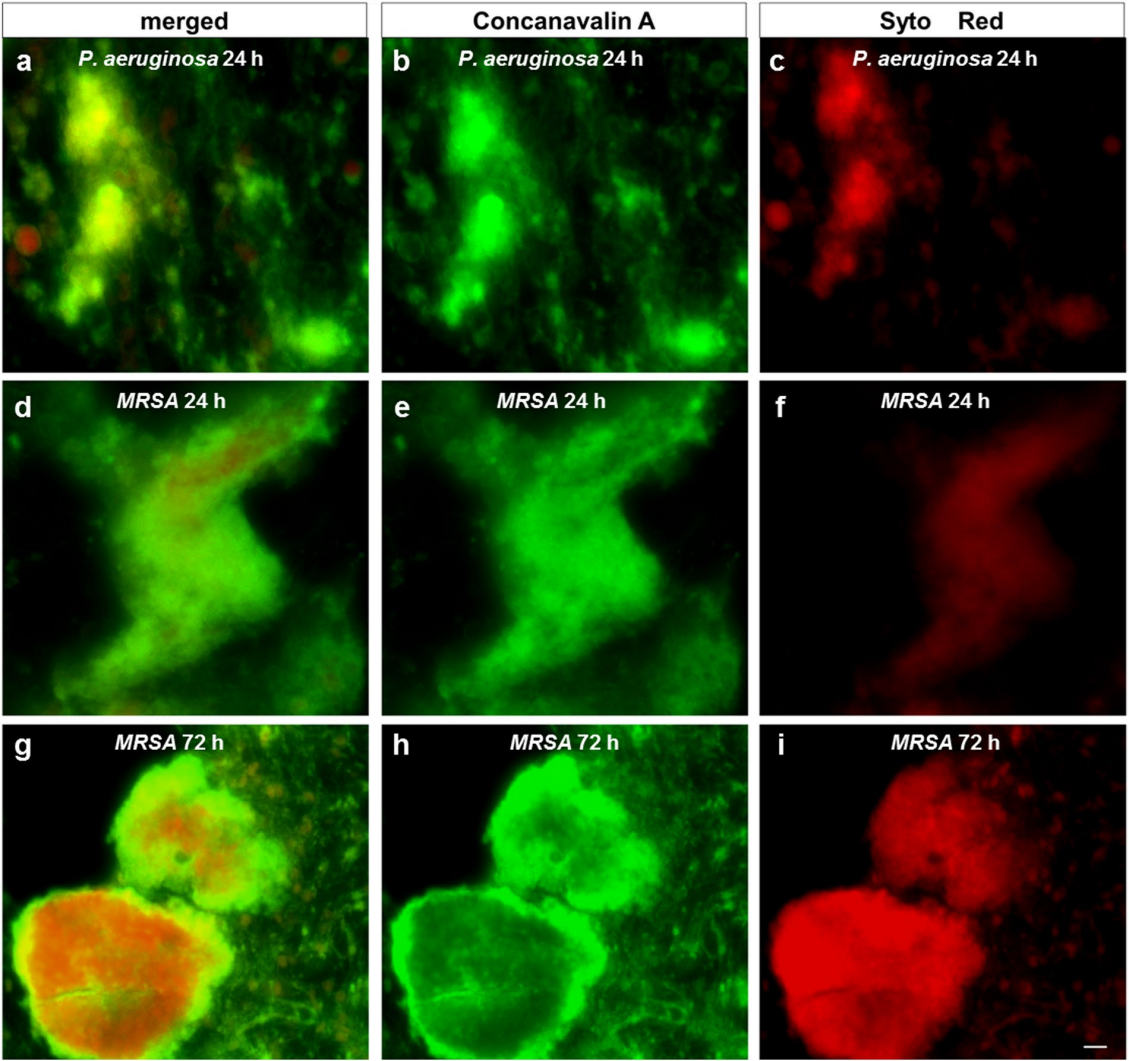
Immunohistochemical staining of carbohydrates in the extracellular matrix. Carbohydrates were detected with FITC-conjugated Con A, cellular and bacterial nucleic acids using SYTO Red staining. **(a–c)** 24 h-maturated biofilm produced by *P. aeruginosa*. **(d–f)** 24 h MRSA-biofilm. **(g–i)** MRSA-biofilm after 72 h of maturation. (scale bar: 20 µm).

**Figure 3:**
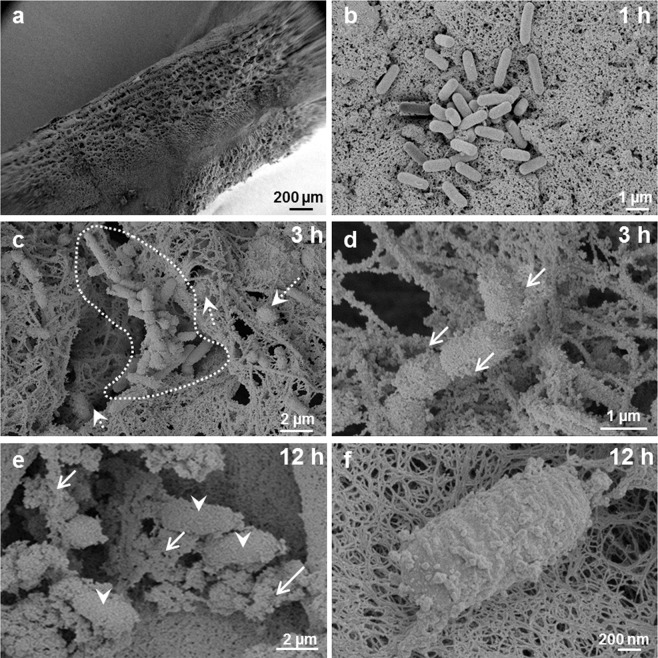
Molecular structure of the hpBIOM. Scanning Electron Microscopy (SEM) data of a *P. aeruginosa* biofilm. **(a)** Breaking edge of the biofilm disc after incubation in liquid nitrogen. **(b)** Bacteria after 1 h incubation. **(c,d)** After 3 h of incubation in the polymerized plasma. Arrows with dotted lines show human cells, dotted circle marks a bacterial colony, arrows with straight lines depict glycokalyx structures. **(e,f)** Glycokalyx development after 12 h of maturation. Arrows with straight lines represent glycokalyx structures, arrow heads depict bacteria. **(f)** Single bacterial cell encapsulated in glycokalyx after 12 h.

**Figure 4:**
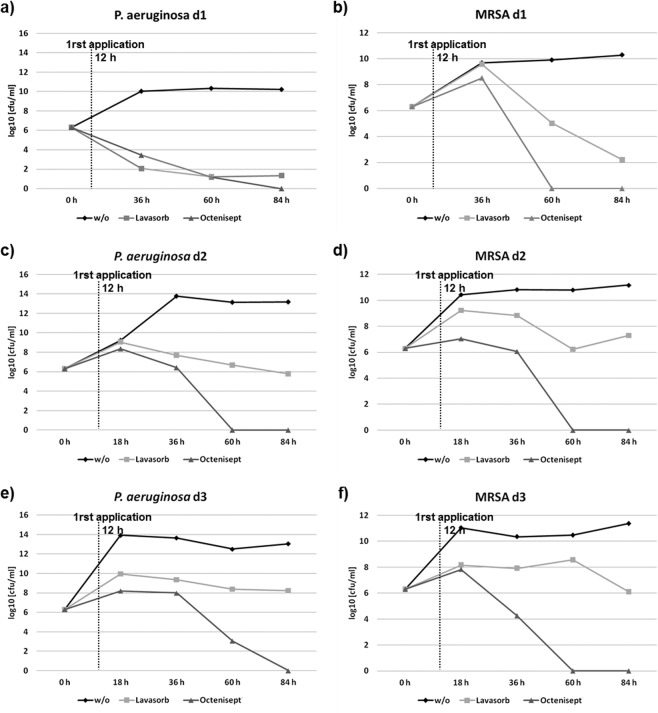
Antimicrobial efficacy of Lavasorb and Octenisept in the 12 h human plasma biofilm. *P. aeruginosa* and MRSA biofilms based on plasma from three independent donors were investigated. The log_10_-reduction rate (cfu/ml) was determined after 6 h, 24 h, 48 h and 72 h. The dotted line reflects the first application of the antiseptics after 12 h of biofilm maturation.

**Figure 5:**
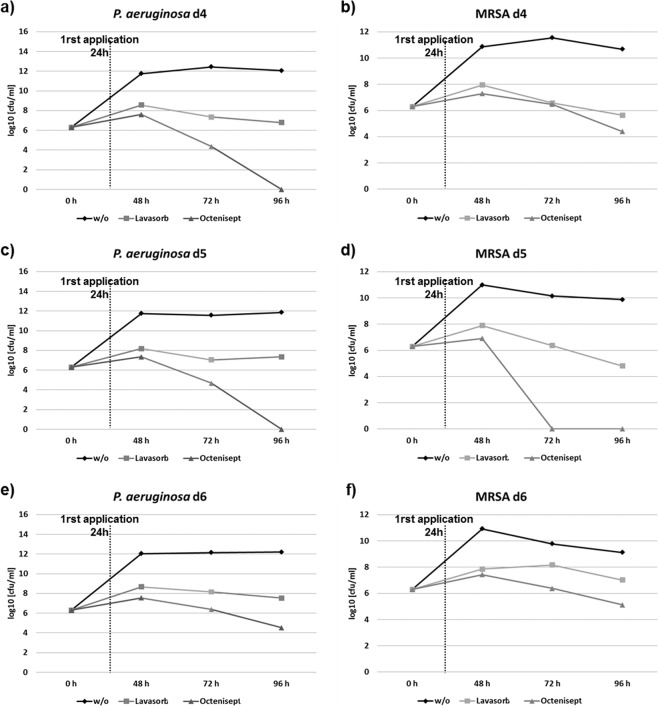
Antimicrobial efficacy of Lavasorb and Octenisept in the 24 h-maturated human plasma biofilm. *P. aeruginosa* and MRSA biofilms based on plasma from three independent donors were investigated. The log_10_-reduction rate (cfu/ml) was determined after 24 h, 48 h and 72 h. The dotted line reflects the first application of the antiseptics after 24 h of biofilm maturation.

In the inflammatory phase of wound healing, vascular permeability increases, leading to plasma exudation and migration of immunocompetent cells into the injured tissue, to restore the microenvironment in terms of pH-regulation cleansing of the tissue and pathogen elimination by the host immune system^31,32^. During biofilm development, bacteria attach to the wound, proliferate and reorganize their surrounding environment to produce the EPS. To closely mimic such wound-biofilm conditions for translational research purposes, our research group developed a biofilm model based on human plasma including the individual buffy coat (containing the cellular immune competence) and integrating selected biofilm-forming pathogens^30^. By means of calcium-induced coagulation, thrombocyte-agglutination and fibrin polymerization, round coagula-like discs with the integrated pathogens are formed (Fig. 1a) which can be further incubated for biofilm maturation. Gram-stainings demonstrated honeycomb-like fibrin structures in the discs, which served as surface for bacterial attachment and scaffold for migrating cells (Fig. 1b,c). Using immunohistological staining with FITC-labeled Concanavlin A, a lectin which selectively binds to carbohydrates, the production of the biofilm-characteristic extracellular polymeric substance (EPS) by *P. aeruginosa* and MRSA was demonstrated within 24 h (Fig. 2). Prolonged incubation (72 h) of the hpBIOM correlated with a maturation of the surrounding EPS matrix (Fig. 2d–i). Additional proof of EPS production, hence biofilm-formation, was provided by using scanning electron microscopy (SEM) to visualize the hpBIOMs glycokalyx at different time-points (Fig. 3). Within the model, bacterial pathogens immediately initiated the production and deposition of glycpolysaccharides (Fig. 3c; 1 h), progressively modulating the surrounding plasma matrix (Fig. 3d,e; 3 h/12 h). Additionally, leukocyte-pathogen-interactions were observed, reflecting a functional active living system (Fig. 3c) within the biofilm model. These observations validated our recently published results^30^. After 12 h of biofilm-maturation, a strong glycokalyx had formed around the individual bacteria (Fig. 3f). The biofilm models disc-like structure remained stable for up to 84 h, however its integrity depending on the individual microorganism or certain combinations. Besides histomorphological visualisation, the main aim of this study was to evaluate the antimicrobial efficacy and activity patterns of the established antimicrobial solutions Octenisept and Lavasorb in the hpBIOM. These antiseptics are usually evaluated based on testing standards (e.g. DIN EN 13727), which are not comparable with the clinical colonization or biofilm-formation in human wounds. Planktonic bacterial suspensions are used, where no adhesion, protective EPS generation or microbial community organization occurs. We used hpBIOMs of *P. aeruginosa* and MRSA. The antiseptics were applied to 12 h- or 24 h- maturated biofilms, representing more matured biofilms with regard to bacterial counts and EPS phenotype. Octenisept and Lavasorb showed different antimicrobial efficacies (Figs. 4, 5): In the 12 h biofilms, the treatment with Lavasorb decreased the bacterial load of *P. aeruginosa* and MRSA compared to the control, however failed to completely eradicate the bacteria within 72 h (Fig. 4a,c,e). This poses a potential risk of re-starting the developmental biofilm-cycle inducing re-infection, especially regarding observed bacterial re-growth between 48 and 72 h in some models (Fig. 4a,b,d). Octenisept proved significantly more effective and managed to completely eradicate both *P. aeruginosa* and MRSA biofilms within 48 h to 72 h of repeated treatment, dependent on the donor (Fig. 4b,d,f). In the more mature 24 h biofilms, the onset of the antimicrobial effect of both antiseptics was delayed (Fig. 5). Nevertheless, the bacterial reduction rate after Lavasorb application was comparable to the results in the less matured biofilms (12 h). Interestingly, in addition to the delayed onset in more matured biofilms (24 h), Octenisept also demonstrated a distinctly reduced bacteriotoxic efficacy on MRSA-produced biofilms. In two of three donors, the bacterial load was not completely eradicated (Fig. 5b,d,f). These results support and verify previous experimental and clinical descriptions of increased biocide tolerance and therapy resistance with progressive biofilm maturation, for instance in biofilms residing in chronic wounds^33,34^.

## Conclusion

This study describes the validation and application of a recently published novel wound biofilm model, comprised of human plasma, thereby more closely resembling the clinical situation of a wound biofilm in humans. The range of applications covers basic science for gaining further knowledge on the development and characteristics of biofilms, implementation of new therapeutic strategies and the validation of already established approaches, such as antiseptic solutions, antibiotics or specific wound dressings. The unique composition closely mimics the actual human wound microenvironment, including the individual immune competence, emphasizing the models advantages regarding research translation into clinical everyday practice. The results of the time-kill-assay using the antiseptic solutions Lavasorb and Octenisept confirmed the importance of these advantages: Manufacturer’s instructions recommend an application time of 1 to 30 minutes. Based on this study result, a significantly longer application time should be taken into account, when dealing with suspected or confirmed biofilm-formation in wounds. Furthermore, antimicrobial efficacy also displayed a certain donor-specificity, suggesting differing effects being based upon individual variations of the wound microenvironment, represented by the individual plasma, in terms of biomolecular composition as well as the immune systems efficiency. In this context the timeliness, representability and transferability of current product evaluation standards used for different aspects such as antimicrobial irrigation, antiseptic and anti-biofilm treatment might have to be revisited and revised to account for repeatedly observed discrepancies. Further studies using the hpBIOM regarding biofilm development and homeostasis as well as translational approaches covering various treatment strategies will provide more detailed insights into the bio-physiology of human wound biofilms as well as best approaches for wound biofilm management.

## Supplementary information

Supplementary Tables.

## Data Availability

The datasets used and/or analysed during the current study are available from the corresponding author on reasonable request.
