# Supplementary material for: Efficacy of antiseptics in a novel 3-dimensional human plasma biofilm model (hpBIOM)
Source: Sci Rep. 2020 Mar 16;10:4792. doi: 10.1038/s41598-020-61728-2 (PMC7075952; doi:10.1038/s41598-020-61728-2)
Supplement: Supplementary file 1 — Supplementary Tables. [file 41598_2020_61728_MOESM1_ESM.doc]

**Efficacy of antiseptics in a novel 3-dimensional
human plasma biofilm model (hpBIOM)**

Besser M^1*^, Dietrich M.^1^, Weber L^1^., Rembe JD^1^, Stuermer EK^2^

^1^ Institute for Translational Wound Research, Centre for Biomedical Education and Research (ZBAF), Witten/Herdecke University, Witten, Germany

^2^Department of Vascular Medicine, University Heart Center, Translational Wound Research, University Medical Center Hamburg-Eppendorf, Hamburg, Germany ^*^Corresponding Author: Manuela Besser, PhD

Witten/Herdecke University

Stockumer Street 10

58453 Witten

Phone: +49 (0) 2 30 2 – 926 332

E-Mail: [Manuela.Besser@uni-wh.de](mailto:Manuela.Besser@uni-wh.de)

Michael Dietrich/ Lea Weber/ Julian-Dario Rembe:

Witten/Herdecke University

Stockumer Street 10

58453 Witten

E-Mail: [michael.dietrich@uni-wh.de](mailto:michael.dietrich@uni-wh.de)

[lea.weber@uni-wh.de](mailto:lea.weber@uni-wh.de)

[julian-dario.rembe@uni-wh.de](mailto:julian-dario.rembe@uni-wh.de)

Ewa-Klara Stuermer: University Medical Center Hamburg-Eppendorf

Martinistr. 52; 20246 Hamburg

[e.stuermer@uke.de](mailto:e.stuermer@uke.de)

Table 1 Time-kill assay with 12 h biofilms

| **Probe** | **Mean** | **SEM** | **Significance compared to w/o** |
| --- | --- | --- | --- |
| D1 P. aeruginosa w/o 36 h  D1 P. aeruginosa w/o 60 h  D1 P. aeruginosa w/o 84 h | 10,01735  10,32064  10,21793 | 0,05709984  0,09714852  0,0707046 |  |
| D1 P. aeruginosa Octenisept 36 h  D1 P. aeruginosa Octenisept 60 h  D1 P. aeruginosa Octenisept 84 h | 3,440049  1,174293  0,000 | 1,73701  1,174293  0,000 | ***  ***  *** |
| D1 P. aeruginosa Lavasorb 36 h  D1 P. aeruginosa Lavasorb 60 h  D1 P. aeruginosa Lavasorb 84 h | 2,066219  1,23299  1,333333 | 2,066219  1,23299  1,333333 | ***  ***  *** |
| D1 MRSA w/o 36 h  D1 MRSA w/o 60 h  D1 MRSA w/o 84 h | 9,668064  9,894382  10,28114 | 0,2319435  0,1892089  0,04812784 |  |
| D1 MRSA Octenisept 36 h  D1 MRSA Octenisept 60 h  D1 MRSA Octenisept 84 h | 8,513078  0,000  0,000 | 0,02111255  0,000  0,000 | ns  ***  *** |
| D1 MRSA Lavasorb 36 h  D1 MRSA Lavasorb 60 h  D1 MRSA Lavasorb 84 h | 9,581171  5,026394  2,218806 | 0,07605648  2,513456  2,218806 | ns  **  *** |
| D2 P. aeruginosa w/o 18 h  D2 P. aeruginosa w/o 36 h  D2 P. aeruginosa w/o 60 h  D2 P. aeruginosa w/o 84 h | 9,070696  13,76769  13,12804  13,1266 | 0,2467874  0,05443876  0,03099669  0,1440051 |  |
| D2 P. aeruginosa Octenisept 18 h  D2 P. aeruginosa Octenisept 36 h  D2 P. aeruginosa Octenisept 60 h  D2 P. aeruginosa Octenisept 84 h | 3,174293  7,606857  6,631594  5,721482 | 3,174293  0,194358  0,1489231  0,1355146 | **  ***  ***  *** |
| D2 P. aeruginosa Lavasorb 18 h  D2 P. aeruginosa Lavasorb 36 h  D2 P. aeruginosa Lavasorb 60 h  D2 P. aeruginosa Lavasorb 84 h | 2,941303  6,240594  0,000  0,000 | 2,941303  0,3123922  0,000  0,000 | **  **  ***  *** |
| D2 MRSA w/o 18 h  D2 MRSA w/o 36 h  D2 MRSA w/o 60 h  D2 MRSA w/o 84 h | 10,2664  10,70187  10,42273  11,01245 | 0,2600179  0,2183008  0,3966033  0,3039967 |  |
| D2 MRSA Octenisept 18 h  D2 MRSA Octenisept 36 h  D2 MRSA Octenisept 60 h  D2 MRSA Octenisept 84 h | 2,507626  6,037748  0,000  0,000 | 2,507626  0,1034087  0,000  0,000 | ***  ***  ***  *** |
| D2 MRSA Lavasorb 18 h  D2 MRSA Lavasorb 36 h  D2 MRSA Lavasorb 60 h  D2 MRSA Lavasorb 84 h | 9,191343  8,732785  6,171775  7,286232 | 0,1151282  0,2384113  0,1741984  0,06685146 | ns  ns  ***  ** |
| D3 P. aeruginosa w/o 18 h  D3 P. aeruginosa w/o 36 h  D3 P. aeruginosa w/o 60 h  D3 P. aeruginosa w/o 84 h | 12,47102  13,63974  12,5133  13,02088 | 0,4555946  0,04766052  0,01253737  0,1167578 |  |
| D3 P. aeruginosa Octenisept 18 h  D3 P. aeruginosa Octenisept 36 h  D3 P. aeruginosa Octenisept 60 h  D3 P. aeruginosa Octenisept 84 h | 5,475323  7,98502  1,174293  0,000 | 2,746862  0,04122954  1,174293  0,000 | ***  ***  ***  *** |
| D3 P. aeruginosa Lavasorb 18 h  D32 P. aeruginosa Lavasorb 36 h  D3 P. aeruginosa Lavasorb 60 h  D3 P. aeruginosa Lavasorb 84 h | 9,526662  9,359246  8,287956  8,198666 | 0,4364281  0,05403322  0,1873734  0,12643 | *  **  **  *** |
| D3 MRSA w/o 18 h  D3 MRSA w/o 36 h  D3 MRSA w/o 60 h  D3 MRSA w/o 84 h | 11,96537  12,40084  12,1217  12,71142 | 0,2600177  0,2183008  0,3966033  0,303997 |  |
| D3 MRSA Octenisept 18 h  D3 MRSA Octenisept 36 h  D3 MRSA Octenisept 60 h  D3 MRSA Octenisept 84 h | 3,07395  7,736721  0,000  0,000 | 3,07395  0,1034083  0,000  0,000 | ***  ***  ***  *** |
| D3 MRSA Lavasorb 18 h  D3 MRSA Lavasorb 36 h  D3 MRSA Lavasorb 60 h  D3 MRSA Lavasorb 84 h | 10,89031  10,43176  7,870747  8,985202 | 0,1151284  0,2384114  0,1741976  0,06685143 | ns  ns  **  ** |

Table 2 Time-kill assay with 24 h biofilms

| **Probe** | **Mean** | **SEM** | **Significance compared to w/o** |
| --- | --- | --- | --- |
| D4 P. aeruginosa w/o 48 h  D4 P. aeruginosa w/o 72 h  D4 P. aeruginosa w/o 96 h | 11,66148  12,28388  12,04177 | 0,1914769  0,2470171  0,08651874 |  |
| D4 P. aeruginosa Octenisept 48 h  D4 P. aeruginosa Octenisept 72 h  D4 P. aeruginosa Octenisept 96 h h | 7,599367  1,615033  0,000 | 0,02587373  1,615033  0,000 | ***  ***  *** |
| D4 P. aeruginosa Lavasorb 48 h  D4 P. aeruginosa Lavasorb 72 h  D4 P. aeruginosa Lavasorb 96 h | 8,572093  7,316977  6,792369 | 0,01098388  0,1071306  0,05165508 | ***  ***  *** |
| D4 MRSA w/o 48 h  D4 MRSA w/o 72 h  D4 MRSA w/o 96 h | 10,74846  10,58045  9,120576 | 0,2262457  0,7204219  1,73377 |  |
| D4 MRSA Octenisept 48 h  D4 MRSA Octenisept 72 h  D4 MRSA Octenisept 96 h | 7,26696  5,947376  4,246788 | 0,09452268  0,4385536  0,2467876 | ***  ***  *** |
| D4 MRSA Lavasorb 48h  D4 MRSA Lavasorb 72 h  D4 MRSA Lavasorb 96 h | 7,914022  6,426291  5,316551 | 0,1269041  0,2362754  0,3853528 | **  ***  *** |
| D5 P. aeruginosa w/o 48 h  D5 P. aeruginosa w/o 72 h  D5 P. aeruginosa w/o 96 h | 11,40246  10,08924  11,86028 | 0,1718829  1,653863  0,03650092 |  |
| D5 P. aeruginosa Octenisept 48 h  D5 P. aeruginosa Octenisept 72 h  D5 P. aeruginosa Octenisept 96 h | 7,233681  4,447475  0,000 | 0,2416734  0,309413  0,000 | ***  ***  *** |
| D5 P. aeruginosa Lavasorb 48 h  D5 P. aeruginosa Lavasorb 72 h  D5 P. aeruginosa Lavasorb 96 h | 8,093585  6,289072  5,019992 | 0,2210114  0,7929247  2,510172 | *  *  *** |
| D5 MRSA w/o 48 h  D5 MRSA w/o 72 h  D5 MRSA w/o 96 h | 10,70879  9,966242  9,683718 | 0,416611  0,3335559  0,3443308 |  |
| D5 MRSA Octenisept 48 h  D5 MRSA Octenisept 72 h  D5 MRSA Octenisept 96 h | 6,875204  0,000  0,000 | 0,1291265  0,000  0,000 | ***  ***  *** |
| D5 MRSA Lavasorb 48h  D5 MRSA Lavasorb 72 h  D5 MRSA Lavasorb 96 h | 7,802346  6,347217  1,759585 | 0,1877267  0,08154158  1,759585 | **  ***  *** |
| D6 P. aeruginosa w/o 48 h  D6 P. aeruginosa w/o 72 h  D6 P. aeruginosa w/o 96 h | 11,95397  12,10978  12,18377 | 0,1919147  0,1364699  0,06775952 |  |
| D6 P. aeruginosa Octenisept 48 h  D6 P. aeruginosa Octenisept 72 h  D6 P. aeruginosa Octenisept 96 h | 7,536956  5,947376  1,666667 | 0,07541473  0,4385536  1,666667 | ***  ***  *** |
| D6 P. aeruginosa Lavasorb 48 h  D6 P. aeruginosa Lavasorb 72 h  D6 P. aeruginosa Lavasorb 96 h | 8,642039  7,816534  7,533824 | 0,1145095  0,3876847  0,02996227 | ***  ***  *** |
| D6 MRSA w/o 48 h  D6 MRSA w/o 72 h  D6 MRSA w/o 96 h | 10,87416  9,56701  9,100344 | 0,1590059  0,218372  0,1003434 |  |
| D6 MRSA Octenisept 48 h  D6 MRSA Octenisept 72 h  D6 MRSA Octenisept 96 h | 7,120118  5,947376  3,489941 | 0,4746532  0,4385536  1,750233 | ***  ***  *** |
| D6 MRSA Lavasorb 48h  D6 MRSA Lavasorb 72 h  D6 MRSA Lavasorb 96 h | 7,81368  7,816534  7,010306 | 0,1438475  0,3876847  0,04964568 | **  ns  * |
